# The Prevalence, Popular Trends, and Associated and Predictive Factors of Non-Obese Fatty Liver Disease

**DOI:** 10.3389/fendo.2021.744710

**Published:** 2021-09-17

**Authors:** Jiang Deng, Yonghong Zhang, Limei Bu, Haitao Shi, Hailing Tang, Shenhao Wang, Qian Wang, Shuangsuo Dang, Ming Li, Zhiyi Han, Xiaolan Lu

**Affiliations:** ^1^Department of Infectious Disease, The Second Affiliated Hospital of Xi’an Jiaotong University, Xi’an, China; ^2^Department of Gastroenterology, Karamay Central Hospital, Karamay City, China; ^3^Department of Gastroenterology, Pudong Hospital, Fudan University, Shanghai, China; ^4^Department of Gastroenterology, The Second Affiliated Hospital of Xi’an Jiaotong University, Xi’an, China; ^5^Division of Gastroenterology, Xi’an Central Hospital, Xi’an, China; ^6^Health Management Department, The Second Affiliated Hospital of Xi’an Jiaotong University, Xi’an, China

**Keywords:** obese, fatty liver disease, prevalence, risk factor, predictor

## Abstract

**Background and Aims:**

There are few studies on non-obese fatty liver disease, the aims of this study was to analyze its prevalence, popular trends, and associated and predictive factors, so as to provide reference for its prevention and treatment.

**Methods:**

Individuals with complete data of body mass index, sex, age, and abdominal ultrasound in Karamay Central Hospital from 2009 to 2016 were selected to analyze the prevalence and popular trends of non-obese fatty liver disease (body mass index <24 kg/m^2^), and associated and predictive factors.

**Results:**

Between 2009 and 2016, a total of 191,555 medical check-ups were included. The prevalence of non-obese fatty liver disease increased from 1.9% to 5.1% among general medical examinants (*P*<0.001), increased from 4.6% to 11.7% in non-obese individuals (*P*<0.001). Compared with the non-obese control group, the levels of age, body mass index, blood pressure, fasting blood glucose, triglycerides, total cholesterol and uric acid in the non-obese fatty liver group were higher (*P*<0. 05). Even among non-obese subjects, elevated body mass index was associated with a 0.63-fold increased risk for non-obese fatty liver disease (*P*<0.001, odds ratio=1.63, 95% confidence interval 1.54-1.72) for every one-unit increase in body mass index. The most common abnormal indicator of non-obese fatty liver disease was elevated triglycerides (44.2%), which was also the best predictor of non-obese fatty liver disease (area under the curve =0.795) in non-obese physical examinators.

**Conclusions:**

The prevalence of non-obese fatty liver disease was high and increasing rapidly in Karamay. Triglycerides is the best predictor of non-obese fatty liver in non-obese physical examinators.

## Introduction

In the past decade, the prevalence of fatty liver disease (FLD) has increased significantly (1–3). In China, non-alcoholic fatty liver disease (NAFLD) is more common than alcoholic FLD, rising from 18% to 29.2% (4), and its prevalence has been consistent with the prevalence of metabolic diseases such as type 2 diabetes mellitus and obesity. According to Chinese standards, the prevalence of being overweight and being obese in adults are 34.3% and 16.4% (5), respectively, and the prevalence of NAFLD in obese individuals is as high as 60%-90% (6). Although the association between high body mass index (BMI) and FLD has received widespread attention (7), there are few studies on non-obese FLD.

Some of the few existing relevant studies are mostly meta-analyses based on the general population, providing a good picture of the global distribution of non-obese FLD (8, 9). However, these studies did not provide the popular trends of non-obese FLD, nor did they propose how to screen it from a large number of people by simple indicators. To address the gaps on popular trends of non-obese FLD over the past decade, describe its characteristics, and identify its indicators in low-resource settings, a long-term observation on a fixed population is necessary.

As such, this study analyzed the laboratory data and physical examination data of Karamay Central Hospital from 2009 to 2016. Different from other studies that focus on the prevalence and outcome of non-obese FLD, this study is the first to analyze the popular trends and predictors of non-obese FLD, with more emphasis on its screening and prevention.

## Materials and Methods

### Materials

A total of 191,555 medical check-ups in Karamay Central Hospital from 2009 to 2016 were analyzed. The city of Karamay, located in northwest China, is an economically developed city that produces oil, with a permanent population of approximately 0.4 million. Karamay Central Hospital is the only large-scale comprehensive hospital in the city with a wide physical examination coverage. It services local residents and employees who consider the medical examiner as a representative of the area.

### Methods

Individuals with complete data of BMI, sex, age, and abdominal ultrasound in Karamay Central Hospital from 2009 to 2016 were selected to analyze the popular trends of non-obese FLD. Non-obese individuals with complete data of BMI, gender, age, abdominal ultrasound, systolic blood pressure (SBP), diastolic blood pressure (DBP), aspartate aminotransferase (AST), alanine aminotransferase (ALT), total bilirubin (TBIL), fasting blood glucose (FBG), triglycerides (TG), total cholesterol (TC), and blood uric acid (UA) were selected for analysis independent influencing and predictive factors of non-obese FLD in 2016. This study was approved by the Ethics Committee of Karamay Central Hospital.

### Diagnostic Criteria

According to China’s criteria for diagnosing overweight and obesity, the diagnosis of FLD in a patient with BMI < 24 kg/m^2^ is considered non-obese FLD. According to the standards of Karamay Central Hospital, ALT >40 U/L, AST >40 U/L, TC >5.69 mmol/L, TG >1.83 mmol/L, FBG >6.2 mmol/L, and TBIL >17.2 U mol/L are elevated in individuals with non-obese FLD.

### Statistical Analysis

Counting data were expressed as ratios, Chi-square exact test was used for comparison. The continuous variables were tested by the t-test or the Mann-Whitney U test. Logistic regression was used for multivariate analysis. The receiver operating characteristic (ROC) curve was used to evaluate the diagnostic efficacy of some meaningful indicators, including BMI, FBG, SBP, TC, ALT and TG, and the differences were considered statistically significant at *P* < 0.05. The IBM SPSS 23.0 Statistic Software (SPSS Inc., Armonk, NY, USA) was used for all statistical analyses.

## Results

### Prevalence of Non-Obese FLD in Physical Examinees From 2009 to 2016

Between 2009 and 2016, a total of 191,555 medical check-ups were included. The prevalence of non-obese FLD increased from 1.9% to 5.1%, showing a 1.7-fold increase (*P*<0.001). More specifically, prevalence increased from 1.9% to 5.4% in males and from 1.8% to 4.8% in females. In general physical examination subjects, the prevalence of non-obese FLD showed no consistent difference between men and women, sometimes higher in men, and sometimes the difference was not statistically significant (**Table 1**).

**Table 1:** Prevalence of non-obese FLD in physical examinants from 2009 to 2016 [%(n+/n)].

Time(Year)	Non-obese FLD/Total	Non-obese FLD/Male	Non-obese FLD/Female	*P*
2009	1.9 (170/8917)	1.9 (117/6046)	1.8 (53/2871)	0.774
2010	2.5 (515/20568)	2.8 (309/10976)	2.1 (206/9592)	0.002
2011	3.7 (884/23977)	3.9 (596/15211)	3.3 (288/8766)	0.012
2012	5.1 (1249/24304)	5.4 (731/13603)	4.8 (518/10701)	0.062
2013	4.6 (1033/22477)	5.1 (639/12436)	3.9 (394/10041)	<0.001
2014	4.2 (1163/27535)	4.5 (768/17093)	3.8 (395/10442)	0.004
2015	3.9 (925/23545)	4.0 (555/14038)	3.9 (370/9507)	0.811
2016	3.9 (1589/40232)	3.8 (889/23338)	4.1 (700/16894)	0.089

non-obese FLD, Non-obese fatty liver disease.

### Prevalence of Non-Obese FLD in Non-Obese Examinees From 2009 to 2016

In the non-obese examinees group, the prevalence of non-obese FLD increased from 2009 to a peak in 2012 before decreasing. From 2009 to 2016, the prevalence of non-obese FLD increased from 4.6% to 11.7%, showing a 1.5-fold increase (*P*<0.001). More specifically, prevalence increased 2.1 times, from 5.8% to 17.8%, in males, and 1.5 times, from 3.1% to 7.9%, in females. The prevalence was statistically higher in males than in females (*P*<0.001) (**Table 2**).

**Table 2:** Prevalence of non-obese FLD in non-obese physical examinees from 2009 to 2016 [%(n+/n)].

Time(Year)	Non-obese FLD/Non-obese examinees	Non-obese FLD/Non-obese Male	Non-obese FLD/Non-obese Female	*P*
2009	4.6 (170/3710)	5.8 (117/2005)	3.1 (53/1705)	<0.001
2010	5.7 (515/9080)	9.4 (309/3299)	3.6 (206/5781)	<0.001
2011	8.4 (884/10489)	12.8 (596/4673)	5.0 (288/5816)	<0.001
2012	11.7 (1249/10659)	17.8 (731/4115)	7.9 (518/6544)	<0.001
2013	10.1 (1033/10185)	16.4 (639/3893)	6.3 (394/6292)	<0.001
2014	9.7 (1163/12019)	14.6 (768/5253)	5.8 (395/6766)	<0.001
2015	8.7 (925/10605)	12.0 (555/4643)	6.2 (370/5962)	<0.001
2016	9.0 (1589/17594)	12.2 (889/7311)	6.8 (700/10283)	<0.001

non-obese FLD, Non-obese fatty liver disease.

### Prevalence of FLD Among Subjects With Different BMIs in 2016 [%(n+/n)]

In 2016, complete data of BMI, sex, age, and abdominal ultrasound was obtained for 40,232 individuals, and the prevalence of FLD was 31.9% (12834/40232), prevalence was higher in males than in females (*P*<0.001), with 39.3% (9171/23338) males and 21.7% (3663/16894) females diagnosed with FLD. The prevalence of FLD in non-obese, overweight, and obese subjects was 9.0%, 39.6%, and 70.8%, respectively, and the differences were statistically significant (*P*<0.001) (**Table 3**).

**Table 3:** Prevalence of FLD among subjects with different BMIs in 2016 [%(n+/n)].

BMI (kg/m^2^)	Total	Male	Female	*P*
<24	9.0% (1589/17594)	12.2% (889/7311)	6.8% (700/10283)	<0.001
24~27.9	39.6% (6053/15302)	41.7% (4466/10718)	34.6% (1587/4584)	<0.001
≥28	70.8% (5192/7336)	71.9% (3816/5309)	67.9% (1376/2027)	0.001

BMI, body mass index.

### Prevalence of Non-Obese FLD Among Non-Obese Physical Examinees of Different Age Groups in 2016 [%(n+/n)]

The prevalence of non-obese FLD were 5.3%, 13.6%, and 16.5% in individuals aged <45 years, 45-55 years, and >55 years, respectively, and the differences were statistically significant (*P*<0.001). Before the age of 55, prevalence was higher in men. However, it was significantly higher in post-menopausal women than in men of the same age group(*P*<0.001) (**Table 4**).

**Table 4:** Prevalence of non-obese FLD among non-obese physical examinees of different age groups in 2016 [%(n+/n)].

Age	Total	Male	Female	*P*
<45 Years	5.3% (557/10487)	9.3% (402/4339)	2.5% (155/6148)	<0.001
45-55 Years	13.6% (660/4846)	18.5% (309/1667)	11.0% (351/3179)	<0.001
>55 Years	16.5% (372/2261)	13.6% (178/1305)	20.3% (194/956)	<0.001

### Comparison of Baseline Data

In 2016, 33,195 individuals with complete data of abdominal ultrasound, age, sex, BMI, SBP, DBP, ALT, FBG, TC, TG, UA, and TBIL had physical examinations in Karamay Central Hospital. Among them, 14,375 were considered non-obese. Compared with the non-obese control group, the levels of age, BMI, systolic blood pressure, diastolic blood pressure, ALT, TBIL, FBG, TG, TC and UA in the non-obese fatty liver group were higher (*P*<0. 05) (**Table 5**).

**Table 5:** Comparison of baseline data of the non-FLD group and the non-obese FLD group among the non-obese physical examinees in 2016.

Characteristics	Fatty liver disease	Non-fatty liver disease	*P*
Total	1284	13091	
Gender			<0.001
male	778 (60.6%)	5449 (41.6%)	
female	506 (39.4%)	7642 (58.4%)	
Age(years)	47.14 ± 11.39	40.31 ± 11.79	<0.001
BMI (kg/m^2^)	22.71 ± 1.10	21.36 ± 1.76	<0.001
FBG(mmol/L)	5.15 (4.73,5.80)	4.83 (4.51,5.19)	<0.001
SBP (mmHg)	128.75 ± 18.18	118.74 ± 15.96	<0.001
DBP (mmHg)	78.85 ± 11.86	73.09 ± 10.84	<0.001
ALT(U/L)	22.10 (16.65,33.00)	15.0 (11.50,21.0)	<0.001
TBIL (u mol/L)	13.70 (10.26,18.05)	13.30 (10.09,17.47)	0.017
TG (mmol/L)	1.70 (1.22,2.37)	0.94 (0.69,1.31)	<0.001
TC(mmol/L)	4.92 ± 0.99	4.45 ± 0.88	<0.001
UA(u mol/L)	332.72 ± 79.55	278.80 ± 73.01	<0.001

BMI, body mass index; FBG, Fasting blood glucose; SBP, Systolic blood pressure; DBP, Diastolic blood pressure; ALT, Alanine transaminase; TBIL, Total bilirubin; TG, Triglycerides; TC, Total cholesterol; UA, Uric acid.

AST/ALT >2 was found in 23 (1.8%) of 1,284 non-obese individuals, and AST >40 U/L was found in eight of them. ALT/AST >1 was detected in 793 individuals (61.8%), and 199 of them had ALT > 40 U/L. Based on these preliminary calculations, the main form of FLD seen in the hospital was NAFLD, with alcoholic FLD rarely diagnosed.

### Characteristics of Non-Obese FLD

Characteristics of 1,284 individuals with non-obese FLD were analyzed, and the abnormal rates, shown in decreasing levels, were as follows: increased TG in 567 individuals (44.2%), increased TBIL in 374 individuals (29.1%), increased SBP in 330 individuals (25.7%), increased TC in 271 individuals (21.1%), increased DBP in 217 individuals (16.9%), increased FBG in 216 individuals (16.8%), increased ALT in 212 individuals (16.5%), and increased UA in 166 individuals (12.9%). Normal TC, TG, SBP, DBP, FBG, and UA were only seen in 380 individuals (29.6%), and only 257 of them had normal ALT and TBIL.

### Logistic Regression Analysis of Non-Obese FLD

Logistic regression analysis showed that sex, age, BMI, FBG, SBP, ALT, TC, TG, and UA were independent influencing factors for the occurrence of non-obese FLD in non-obese physical examinees in Karamay Central Hospital in 2016 (*P*<0. 05). In non-obese individuals, elevated BMI was associated with a 0.63-fold increased risk for non-obese FLD (*P*<0. 001, odds ratio [OR]=1.63, 95% confidence interval [CI]: 1.54-1.72) for every one-unit increase in BMI (**Table 6**).

**Table 6:** Logistic regression analysis of non-obese fatty liver disease in 2016 non-obese physical examination subjects.

Characteristics	B	*P*	OR	95% CI for OR
Gender(Male/female)	0.48	<0.001	1.62	1.38 1.91
Age(Years)	0.03	<0.001	1.03	1.02 1.03
BMI(kg/m^2^)	0.49	<0.001	1.63	1.54 1.72
FBG(mmol/L)	0.14	<0.001	1.15	1.11 1.20
SBP(mmHg)	0.01	0.017	1.01	1.00 1.01
DBP(mmHg)	0	0.324	1.00	1.00 1.01
ALT(U/L)	0.01	<0.001	1.01	1.01 1.01
TBIL(u mol/L)	0	0.641	1.00	0.99 1.01
TC(mmol/L)	0.13	0.001	1.13	1.06 1.22
TG(mmol/L)	0.42	<0.001	1.53	1.43 1.63
UA(u mol/L)	0.01	<0.001	1.01	1.01 1.01
Constant	-19.72	<0.001	0	- -

BMI, body mass index; FBG, Fasting blood glucose; SBP, Systolic blood pressure; DBP, Diastolic blood pressure; ALT, Alanine transaminase; TBIL, Total bilirubin; TG, Triglycerides; TC, Total cholesterol; UA, Uric acid.

### The ROC Curve Was Used to Evaluate the Diagnostic Efficacy of the Indices in the Diagnosis of Non-Obese FLD

BMI, FBG, SBP, ALT, TC, and TG were analyzed by the ROC curve. Results showed that the area under the curve (AUC) value of triglycerides was the highest, which was 0.795, illustrating that TG had the best diagnostic efficiency. The maximum Youden index was taken as the critical value, and the sensitivity and specificity determined at this time were 74.1% and 72.2%, respectively. In addition, the AUC value of TG combined with ALT was 0.815.

**Figure 1:**
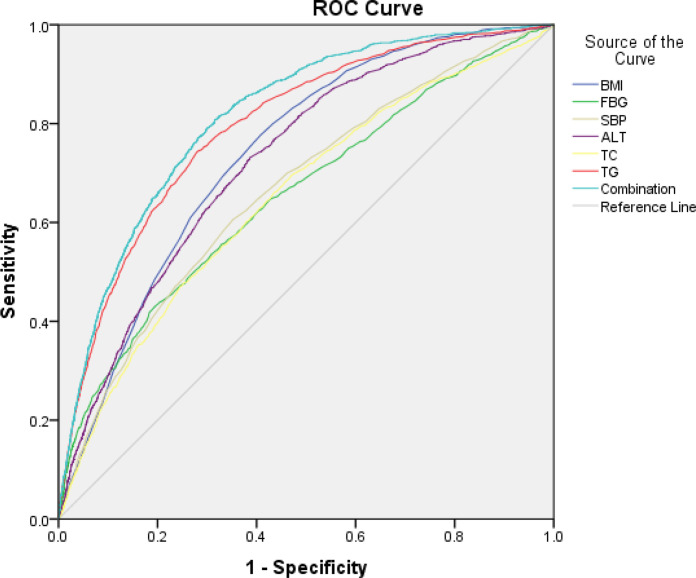
The ROC curve was used to evaluate the diagnostic efficacy of index in the diagnosis of non-obese FLD.

Therefore, considering the absence of physical examination and/or imaging and based on a large volume of physical indicators analyzed, TG is the best indicator in screening non-obese FLD among non-obese physical examinees. **Table 7** and **Figure 1**.

**Table 7:** The ROC curve was used to evaluate the diagnostic efficacy of index in the diagnosis of non-obese fatty liver disease.

Variable	Area	*Std*	*P*	95% CI for OR	Cut-off point	Sensitivity	Specificity
BMI(kg/m^2^)	0.739	0.006	<0.001.	0.727 0.752	22.05	0.777	0.591
FBG(mmol/L)	0.655	0.008	<0.001	0.638 0.671	5.32	0.427	0.808
SBP(mmHg)	0.665	0.008	<0.001	0.650 0.681	122.5	0.607	0.647
ALT(U/L)	0.727	0.007	<0.001	0.713 0.740	17.06	0.732	0.613
TC(mmol/L)	0.650	0.008	<0.001	0.634 0.666	4.44	0.695	0.530
TG(mmol/L)	0.795	0.006	<0.001	0.782 0.807	1.25	0.741	0.722
TG+ALT	0.815	0.006	<0.001	0.803 0.826	–	–	–

BMI, body mass index; FBG, Fasting blood glucose; SBP, Systolic blood pressure; ALT, Alanine transaminase; TG, Triglycerides; TC, Total cholesterol.

## Discussion

This study shows that, between 2009 and 2016, the prevalence of non-obese FLD increased from 1.9% to 5.1% in general medical examiner, the prevalence of non-obese FLD increased from 4.6% to 11.7% in the non-obese examinees group. A meta-analysis from China showed that the prevalence of NAFLD in the non-obese population was 10.8% (95% CI: 9.0%-12.6%) (4). Another meta-analysis from 14 countries showed that the global prevalence of lean NAFLD was 4.1% (95% CI: 3.4%-4.8%) in the general population and 9.7% (95% CI: 7.7%-11.8%) in the lean population (8). These figures are consistent with the results of our study. In 2020, a meta-analysis covering 24 countries and regions showed that the overall proportion of non-obese NAFLD in the global NAFLD population was 40.8% (95% CI: 36.6-45.1). In addition, the prevalence of non-obese NAFLD in the general population was 12.1% (95% CI: 9.3-15.6) (9), which is much higher than the prevalence determined in other studies (1.9%-5.1%). The differences may be related to race, genetic polymorphism (PNPLA3, rs738409, etc.), regional differences, differences in diagnostic criteria, and other factors. Despite having the same BMIs, distribution of body fat varies by region and ethnicity; Asians store more visceral or abdominal fat at lower BMIs, which puts them at a higher risk for the disease (10). There is substantial evidence that Chinese may have a higher percentage of body fat and therefore have both a higher cardiovascular risk and all-cause mortality than Caucasians at the same BMIs (5).

The prevalence of lean FLD varies greatly among different ethnic groups and regions. In the overall population, the prevalence of lean NAFLD is the highest in Asia (4.8%), which is followed by Oceania (3.5%), North America (3.1%), and Europe (2.2%). The highest prevalence is seen in China (5.5%) (95% *CI*: 2.5-8.5%) and the lowest prevalence is in the United States (3.1%) (95% *CI*: 2.3-3.8%) (8). Heterogeneity in study area, study time, and study subjects are some factors that may cause these differences. Compared to the prevalence of lean NAFLD based on health screening (4.5%, 95% *CI*: 3.9 5.2%) and community-based studies (3.0%, 95% *CI*: 0.7 5.3%), population-based studies (5.7%, 95% *CI*: 3.6 7.7%) showed the highest prevalence (8).

Non-obese FLD can occur in non-obese individuals who have a normal BMI but have recently gained weight or increased their waist circumference. Some obese people lose a lot of weight too fast, leading to a large amount of fat decomposition, as the liver’s ability to process free fatty acids is limited, a large amount of fat accumulates in the liver, resulting in FLD. Obese people may also take a vegetarian diet to lose weight, reducing protein intake and subsequently lacking apolipoprotein, triglycerides cannot be transferred out of the liver without apolipoprotein, leading to the formation of malnutrition FLD (6). Other causes of FLD include uneven distribution of body fat, high visceral fat, and metabolic disorders.

BMI alone cannot be used as a preliminary screening index. Compared to subcutaneous fat and BMI, visceral obesity is a better indicator for developing NAFLD in non-obese individuals. Additionally, waist-to-hip ratio may actually be more likely to identify abdominal obesity than BMI. Besides these, neck circumference and body fat analysis can also be used to screen non-obese FLD (11). There are also studies that showed how the sagittal abdominal diameter (SAD) can reflect the ability of abdominal fat more accurately than waist circumference and BMI, especially in young and non-obese individuals. SAD exhibited a stronger correlation with risk factors of metabolic syndrome compared to waist circumference, waist-to-hip ratio, and BMI. As such, the combined diagnostic power of multiple indicators may be higher than that of a single indicator (12–14).

The risk for FLD differs among age groups and sex. Genetic factors and excessive nutrition are important reasons in diagnosing FLD in children (15). For adults, FLD is affected by hormones, lifestyle, work stress, genetics, and other factors (4). The prevalence of FLD in women increased significantly across time, the reasons may be related to the decline of ovarian function, the decrease of estrogen level, and the increased risk of metabolic diseases. Conversely, this increasing trend is not completely consistent in men. With increasing age, the body’s hormone level changes, activity decreases, and lipid metabolism function declines. Current research makes it clear that as age increases, airframe fat turnover evidently drops. In fact, in a male not following an abstinent diet, weight increases by 20% on average (16, 17). This may also be related to work and life stress.

Compared with the non-obese control group, the levels of age, BMI, systolic blood pressure, diastolic blood pressure, ALT, TBIL, FBG, TG, TC and UA in the non-obese fatty liver group were higher (*P*<0. 05). The results are consistent with previous studies based on the general population and non-obese individuals (4, 8, 18, 19). NAFLD is a manifestation of metabolic syndrome involving the liver (6), and metabolic abnormalities therefore increase the risk of FLD. For the general population, metabolic abnormalities such as high BMI and triglycerides are the important independent risk factors associated with the occurrence of FLD. Although people with lean NAFLD may have better metabolic syndrome-related indicators and a lower incidence of metabolic complications compared to overweight and obese individuals with NAFLD (8, 20), lean FLD remains to be associated with a higher risk for metabolic disorders (21, 22). This study showed that in 1,284 people with non-obese FLD, the most common abnormal metabolic index was high triglycerides (44.2%), and its AUC value was the highest. Combined the results of logistic regression and ROC curve, it is difficult to identify FLD based on BMI in non-obese physical examinees who lacked diagnostic equipment or did not have access to imaging. Additionally, triglycerides appeared to be the best predictor of diagnosing FLD in non-obese physical examinees.

In addition to metabolic and genetic factors, muscle atrophy and loss of muscle strength (23, 24), unhealthy eating patterns (i.e., High cholesterol and fructose intake) (25), and changes in intestinal flora are also important influencing factors that do not only promote the occurrence of non-obese FLD but also aggravate its progression. Studies have shown that the decrease of butyrate-producing *Eubacterium* may play an important role in the development of NAFLD in non-obese individuals (26). In non-obese individuals, *Ruminococcaceae* and *Veillonellaceae* were the predominant microbiota associated with severe hepatic fibrosis (27). In a prospective cohort study of 307 individuals from Hong Kong, Leung et al, reported that non-obese individuals (23.5%) had a lower incidence of metabolic syndrome and a lower NAFLD activity score (28). Although non-obese NAFLD individuals have healthier metabolic profiles and less advanced fibrosis, their prognosis may be worse than obese NAFLD patients (25, 29). Results of a cohort study of 646 patients with biopsy-confirmed NAFLD showed that lean NAFLD patients were older, and had lower transaminase levels, lower fibrosis stage, and lower prevalence of non-alcoholic steatohepatitis than those with higher BMI. At a mean follow-up time of 19.9 years (0.4-40 years), compared with overweight patients, patients with lean NAFLD had no increased risk for overall mortality (hazard ratio [HR]=1.06, *P*=0.73) but had an increased risk for severe liver disease (HR=2.69, *P*=0.007) (20). Additionally, cardiovascular events are a major factor affecting the prognosis of patients with NAFLD, and all patients with NAFLD should therefore be assessed for the risk of cardiovascular events (6) to reduce the incidence of complications and adverse outcomes.

This was a large single-center retrospective study. Subjective data, such as history of alcohol consumption and diabetes, are hard to come by. Objective data such as fasting glucose and BMI were used to reflect the baseline data of patients. Unlike other studies, this study did not follow clinical outcomes, nor were visceral fat levels measured. In China and some other places, the main cause of end-stage liver disease is viral liver disease—not FLD (6), there were only 14 cases of cirrhosis and 4 cases of liver fibrosis among the 40,232 people who underwent ultrasound examination in 2016. Moreover, early FLD is a curable disease, more research should focus on attention, screening, prevention and treatment (22, 30). Large-scale measurements of visceral fat to determine the risk of developing non-obese FLD are difficult and impractical in terms of resources, which can instead be allocated to the prevention and treatment of non-obese FLD for better outcomes. Therefore, the focus of this study is to analyze the popular trends of non-obese FLD to attract people’s attention, and then analyze the characteristics of non-obese FLD, and find a simple but effective screening method, which can be applied to a large number of people.

## Data Availability Statement

The original contributions presented in the study are included in the article/supplementary material. Further inquiries can be directed to the corresponding author.

## Ethics Statement

This study was approved by the Ethics Committee of Karamay Central Hospital.

## Author Contributions

JD, ZH, and XL conceived and designed the study. JD, YZ, LB, HS, HT, SD, and ML collected the data. JD analyzed the data. All authors contributed to the article and approved the submitted version. JD, ML, ZH, and XL wrote the manuscript.

## Funding

1. Key Discipline Construction Project of Pudong Health and Family Planning Commission of Shanghai (Grant No. PWZxk 2017-27); 2. This study was supported by National Key Research and Development Program of China during the 13th Five-Year Plan Period (2018YFC1311504); 3.This work was supported by Talents Training Program of Pudong Hospital affiliated to Fudan University (YJRCJJ201801).

## Conflict of Interest

The authors declare that the research was conducted in the absence of any commercial or financial relationships that could be construed as a potential conflict of interest.

## Publisher’s Note

All claims expressed in this article are solely those of the authors and do not necessarily represent those of their affiliated organizations, or those of the publisher, the editors and the reviewers. Any product that may be evaluated in this article, or claim that may be made by its manufacturer, is not guaranteed or endorsed by the publisher.

## Abbreviations

FLD, fatty liver disease; NAFLD, non-alcoholic fatty liver disease; BMI, body mass index; SBP, systolic blood pressure; DBP, diastolic blood pressure; AST, aspartate aminotransferase; ALT, alanine aminotransferase; TBIL, total bilirubin; FPG,fasting blood glucose; TG, triglycerides; TC, total cholesterol; UA,uric acid; ROC,receiver operating characteristic curve; OR, odds ratio; AUC, area under the curve; CI, confidence interval; SAD, sagittal abdominal diameter; HR, hazard ratio.
